# The Impact of COVID-19 Restrictions on the Healthy Eating and Movement Behaviors of 0–12-Year-Old Children in Western Sydney, Australia

**DOI:** 10.3389/fpubh.2022.841178

**Published:** 2022-05-24

**Authors:** Janelle McNicholas, Megan L. Hammersley, Stacey Hopkins, Sarah McDermott, Jennifer Plaskett

**Affiliations:** ^1^Centre for Population Health, Western Sydney Local Health District, Sydney, NSW, Australia; ^2^Early Start, Faculty of the Arts, Social Sciences and Humanities, University of Wollongong, Wollongong, NSW, Australia; ^3^School of Health and Society, Faculty of the Arts, Social Sciences and Humanities, University of Wollongong, Wollongong, NSW, Australia; ^4^Illawarra Health and Medical Research Institute, University of Wollongong, Wollongong, NSW, Australia

**Keywords:** coronavirus, lockdown, lifestyle behaviors, physical activity, screen time, diet, nutrition, food security

## Abstract

This study examined effects of COVID-19 restrictions in early 2020 on movement (physical activity, sedentary behavior and sleep) and healthy eating behaviors in families with 0–12-year-old children in western Sydney, Australia. A total of 1,371 parents completed an online survey about changes in children's and families' food intake and movement behaviors. There was an increase in sedentary screen use by children (4.18/5.00) and families (3.91/5.00) and a slight increase in reported physical activity (3.56/5.00), amount of food consumed (3.58/5.00) and meals and snacks eaten (3.69/5.00) during the height of the COVID-19 restrictions compared to before. There was little change in reported sleep (3.17/5.00). Lower socioeconomic families were disproportionately affected, with greater increases in unhealthy eating (t = 2.739, *P* = 0.06), lower levels of improvement in physical activity, such as walking and cycling (t = −7.521, *P* < 0.001) and outdoor activity (t = 5.415, *p* < 0.001), and higher increases in family sedentary behavior (t = 2.313, *P* = 0.021). Therefore, even short periods of restrictions can result in detrimental health behavior changes. Such changes could become entrenched leading to increased risk of lifestyle diseases. Programmatic and policy strategies should be geared toward promoting healthy movement behaviors, focusing on families of lower socioeconomic status to ensure the pandemic does not widen an existing gap.

## Introduction

The novel coronavirus disease 2019 (COVID-19) pandemic has had a significant impact on everyday life worldwide. Since the declaration of the global pandemic and the first national cases, Australia has had a total of just over 200,000 confirmed cases and 1,968 deaths (from March 2000 through to November 2021) (1). Australia has been faring relatively well compared to the rest of the world, with one of the lowest death rates (7.78 per 100 000 population) (2).

In response to this pandemic, in March 2020, each state in Australia implemented several approaches to reduce the health and economic impact of COVID-19. In New South Wales (NSW), schools and childcare services only remained open for the children of essential workers (hospital staff, teachers, police staff etc), workplaces implemented work-from-home strategies and several restrictions were imposed on the community as cases increased, such as number of visitors permitted in households and outdoor areas, the closure of restaurants, cafes, amusement parks, play centers, gyms, playgrounds, cinemas, and the cancellation of team sports/children's group activities. The height of these restrictions in NSW in 2020 occurred between March-May for a period of 62 days. However, unlike other countries and states within Australia, during this period of restrictions in NSW, there were no restrictions on the amount of time people were permitted outside for exercise. Studies conducted during the COVID-19 pandemic world-wide have shown that children had decreased physical activity, increased screen time and increased unhealthy eating and snacks (3–8). It is anticipated that COVID-19 restrictions in Australia during 2020 likely had a lesser impact on child health behaviors than what has been observed globally given the restrictions at this time for most Australian states except Victoria, was for a short period compared to the rest of the world. However, to date, there are limited data on the impact of the Australian restrictions on the health behaviors of children and families.

During the height of the 2020 COVID-19 restrictions, more than three quarters of Australian households with children kept their children home from school or childcare. While there are currently limited data available on child health behaviors in Australia during this time, over 20% of adults reported eating more discretionary foods and almost 60% indicated that they were spending more time in front of screens (9). As many children were at home during this period, it is likely that there were similar changes in these behaviors among children. There were however some positive changes seen over this time regarding fruit and vegetable consumption and more cooking at home (9). However, with an increasing number of adults losing work, many households were likely under greater financial pressure and struggling to provide healthy foods for their families.

Understanding the association between COVID-19 restrictions and the health behaviors of children and families is important in guiding decision-making among policymakers, and in educating parents and health professionals. Even in short periods of heightened restrictions, deterioration in healthy lifestyle behaviors has the potential to be embedded into usual family routines and have a long-term impact on dietary intake, movement behaviors and weight status (3). The aim of this study was to identify changes in child and family eating and movement behaviors (including physical activity, sedentary behavior and sleep) in families from western Sydney with children 0–12-years of age during the height of the COVID-19 restrictions in 2020.

## Methods

### Data Collection

This cross-sectional study was conducted in Western Sydney Local Health District (WSLHD). Western Sydney is a region of metropolitan Sydney (the largest city in Australia, and the capital city of the state of NSW). In NSW, the health system is divided up geographically into 15 Local Health Districts (LHDs), WSLHD being one of the 15 regions. The WSLHD has a total population of 946 000 people (12.5% of the NSW population), with 196 259 in the 0–14 years age group (20.8%), a higher proportion of children when compared to NSW overall (18.5%). There are 172 186 households with children under the age of 15 in the WSLHD, representing 70.3% of all households in the jurisdiction, also higher than the overall proportion of 61.8% in NSW (10). A survey was distributed widely to parents in this health jurisdiction via established networks of the WSLHD health promotion team. Specifically, it was sent to 1,040 schools, 527 early childhood education and care services, a Western Sydney LHD newsletter, and was posted on the Healthy Kids Western Sydney website. As the survey was distributed through these avenues, it was not possible to determine the number of families that ultimately received the survey. The survey sought to ascertain changes in dietary intake and movement (physical activity, sedentary behavior and sleep) behaviors of young children (0–12- years of age) and families during the period of the height of the 2020 COVID-19 restrictions in Sydney, Australia (during late March to mid-May) compared to before these restrictions were imposed. The online survey was distributed to parents via email in June 2020, following the easing of restrictions in NSW. Parents were asked to answer the child-related questions referring to the eldest child in the household between the ages of 0–12 years. The survey took approximately 15 min to complete. To thank participants for their time, they were offered the opportunity to go into a draw to win a $100 gift card. The survey was conducted as part of a quality assurance project (2008-03), approved by the Westmead Scientific Advisory QA Committee and the Secretary of the WSLHD Human Research Ethics Committee. The reporting of this research aligns with the STROBE statement (11).

### Measures

Movement behavior questions for the survey were derived from a recent survey designed and conducted by partipACTION in Canada on changes in movement behaviors during the COVID-19 outbreak (3) which had been tested for reliability. Dietary intake questions were based on a survey conducted during the New Zealand COVID-19 lockdown in 2020, through the Growing Up in New Zealand COVID-19 Wellbeing Survey (12). Additional questions on food security were also included which were developed specifically for this survey.

All survey questions are outlined in Table 1. Child movement behavior questions enquired about changes in walking and bike riding, physical activity, sport and play in their backyard, park, local street and sporting field, household chores, screen time, social media use, non-screen indoor activities, and sleep. Family movement behavior questions enquired about changes in physical activity and sedentary behavior. Parents were asked to respond to these questions on a 5-point Likert scale (1 = a lot less to 5 = a lot more). Child dietary intake questions enquired about increases in overall food intake, meals and snacks, fruit, vegetables, discretionary foods and beverages, water, and food variety. Parents were asked to respond to these questions on a 5-point Likert scale (1 = totally disagree to 5 = totally agree). Family eating practice questions enquired about home-cooked meals, takeaway meals, and meals eaten together at the dinner table. Parents were asked to respond to these questions on a 5-point Likert scale (1 = a lot less to 5 = a lot more). Regarding food security, parents were asked if there were times in the last 12 months that they ran out of food (yes/no/do not wish to respond), and if this had changed since the COVID-19 outbreak (stayed the same/less able to afford food/more able to afford food/unsure/do not wish to respond). Additionally, an open response question asked parents if there were any behaviors that their child/ren and/or family had done more or less of during the COVID restriction period that they would like to maintain or continue. Parents and carers were asked to provide information on demographic characteristics including postcode (Australian equivalent to zip code), number of adults and children aged 0–12 in the home, type of dwelling, proximity to a local park, number of days children attended preschool, childcare or school prior to and during the height of the COVID-19 restrictions. Postcode was used as a proxy for socioeconomic status (SES), based on the Australian Bureau of Statistics' Socioeconomic Indexes for Areas.

**Table 1:** Items from survey.

**Question**	**Response**
**Demographic questions**	
1. What is your postcode	Numeric
2. How many children live in your home?	Numeric
3. How many children aged 0–12 years live in your home?	Numeric
4. Do you live in a…	House, apartment/unit, villa/townhouse, duplex, other
5. How many minutes does it take you to walk to your local park?	Numeric (min)
**Food security**	
6. Over the last 12 months, were there times that you ran out of food and couldn't afford to buy more?	Yes, no, don't know, don't wish to respond
7. Since the COVID19 outbreak, how has your ability to afford food changed?	It has stayed the same, Less able to afford food, more able to afford food, unsure, do not wish to respond
**School/pre-school/childcare attendance**	
8. Please list the ages of the children in your family	Numeric
9. On average, how many days each week do your child/ren aged 0-12 years usually attend preschool, childcare or school prior to the COVID-19 outbreak	Numeric
10. On average, how many days each week did your child/ren attend during the height of COVID-19 restrictions (when parents were encouraged to keep their children home from school – from late March to mid-May)?	Numeric
**Child eating behaviours**	
11. During the height of COVID-19 restrictions (late-March to mid-May 2020), thinking of your eldest child aged 0-12 years, did your child:	5-point Likert scale (from totally disagree to totally agree)
a. Eat more food than before	
b. Eat more meals and snacks during the day than before	
c. Eat more fruit than before	
d. Eat more vegetables than before	
e. Eat more potato chips, chocolate, biscuits or cake than before	
f. Drink more soft drink, cordial or juice than before	
g. Drink more water than before	
h. Eat a greater variety (different types) of food than before	
**Child movement behaviours**	
12. During the height of COVID-19 restrictions (late-March to mid-May), comparted to before the COVID-19 outbreak, did your eldest child (aged 0-12 years) do the following activities more or less	5-point Likert scale (from a lot less to a lot more)
a. Walked around the neighbourhood?	
b. Rode their bike or scooter around the neighbourhood?	
c. Did physical activities, sport or played in their backyard or garden?	
d. Did physical activities, sport or played on the local street?	
e. Did physical activities, sport or played in the local park?	
f. Did physical activities, sport or played in local playing field?	
g. Did physical activities inside?	
h. Did household chores (e.g. cleaning, gardening)?	
i. Watched TV, movies, used the computer, tablets or smart phones for leisure?	
j. Used social media?	
k. Did other activities involving sitting down e.g. puzzles, reading, drawing and not in front of screens?	
l. Slept?	
**Family movement behaviours**	
13. During the height of the COVID-19 restrictions (late-March to mid-May), compared to before the COVID-19 outbreak, on average, did your family spend less or more…	5-point Likert scale (from a lot less to a lot more)
a. Time in physical activity?	
b. Time in sedentary activities such as watching TV, using consoles and devices such as tablets and smart phones?	
**Family eating behaviours**	
14. During the height of the COVID-19 restrictions (late-March to mid-May), compared to before the COVID-19 outbreak, on average, did your family eat…	5-point Likert scale (from a lot less to a lot more)
a. Home cooked meals	
b. Takeaway meals	
c. Meals together at the dinner table	
15. Are there any behaviours that your child/ren AND/OR family has done more OR less of during the COVID period that you would like to maintain or continue?	Open text

### Statistical Analysis

Means and standard deviations, and percentages were calculated for variables. For the purposes of statistical analyses, some variables were combined into a score. Eat more food, and eat more snacks were combined, producing an “eat more” score out of 5. Eat more fruit, and eat more vegetables were combined to create a “eat healthier foods” score out of 5. Eat more potato chips, chocolate biscuits and cake, and drink more soft drink, cordial or juice were combined to create a “eat unhealthier foods” score out of 5. Walking and bike riding were combined to create a “walking and bike riding” score out of 5, and physical activity, sport or play in: the backyard/garden, local street, local park, and local playing field, were combined to create a “physical activity” score out of 5. Independent *t*-tests were used to determine differences in movement and eating behavior variables between age group categories (<5 years and 5–12 years). Independent *t-*tests were also conducted to determine differences in movement and eating behavior variables based on SEIFA (measure of socioeconomic status determined by residential postcode) deciles of 1–5 (least disadvantaged), and 6–10 (most disadvantaged). ANOVA tests were conducted to determine differences in movement behaviors between type of dwelling that the child resided in (house; apartment/unit; or villa/townhouse/duplex/other). Spearman correlation analyses were conducted to determine associations between changes in movement and eating behaviors. Chi square tests were used to determine differences between lower and higher income groups in regard to food security issues both before and during the COVID-19 restrictions. All statistical analyses were conducted in SPSS v 25 (SPSS In, Chicago IL, USA).

### Qualitative Analysis

Two researchers reviewed the responses to the one open-ended question of the survey that asked about any behaviors that children/family had done more or less of that they would like to maintain. Both researchers first read all responses independently before beginning to code. The first 100 responses were then independently coded by each researcher and compared in order to establish inter-coder reliability and consistency. An 87% consistency was achieved; discrepancies were due to differences in code labels (e.g., spending time with family vs. family time) rather than a misclassification by assessors. The responses were then grouped into themes, which were categorized as participating in behaviors “more” or “less”. Initial themes were reviewed and edited (for example renamed or collapsed) until each theme described a unique aspect of the data.

## Results

### Demographic Characteristics

Survey responses were received from 1,371 parents. Demographic data from this sample are displayed in Table 2. The mean age of the eldest child was 6.75 (SD 3.22), the mean number of adults per household was 2.27 (SD 0.79) and the mean number of children was 1.91 (SD 0.81). The majority of participants lived in houses (71.5%), compared to approximately 65% of people in WSLHD (10). There was a broad range of socioeconomic profiles, based on postcode of residence, a relatively high proportion (75%) of families in most advantaged socioeconomic SEIFA deciles (6–10).

**Table 2:** Demographics of participants (*n* = 1,371).

**Variable**	
Age of eldest child, M(SD)	6.75 (3.22)
Number of adults in household, M(SD)	2.27 (0.79)
Number of children in household, M(SD)	1.91 (0.81)
**Type of residence** ***n*** **(%)**	
House	968 (71.5%)
Apartment or unit	162 (12.0%)
Villa, townhouse, duplex or other	224 (16.5%)
**SEIFA Decile** ***n*** **(%)**	
1–5	340 (24.8%)
6–10	1,031 (75.2%)
Proximity to local park (minutes), M(SD)	7.5 (3.22)
**Food security–ran out of food in last 12 months**, ***n*** **(%)**	
Yes	102 (7.4%)
No	1,135 (82.8%)
**Food security since COVID-19**, ***n*** **(%)**	
Less able to afford food	254 (18.5%)
Stayed the same	914 (66.7%)
More able to afford food	52 (3.8%)
Number of days per week in child care or school for eldest child prior to COVID-19 restrictions M(SD)	4.08 (1.58)
Number of days per week in child care or school for eldest child during height of COVID-19 restrictions M(SD)	0.94 (1.72)

### Changes in Eating and Movement Behaviors

**Figure 1:**
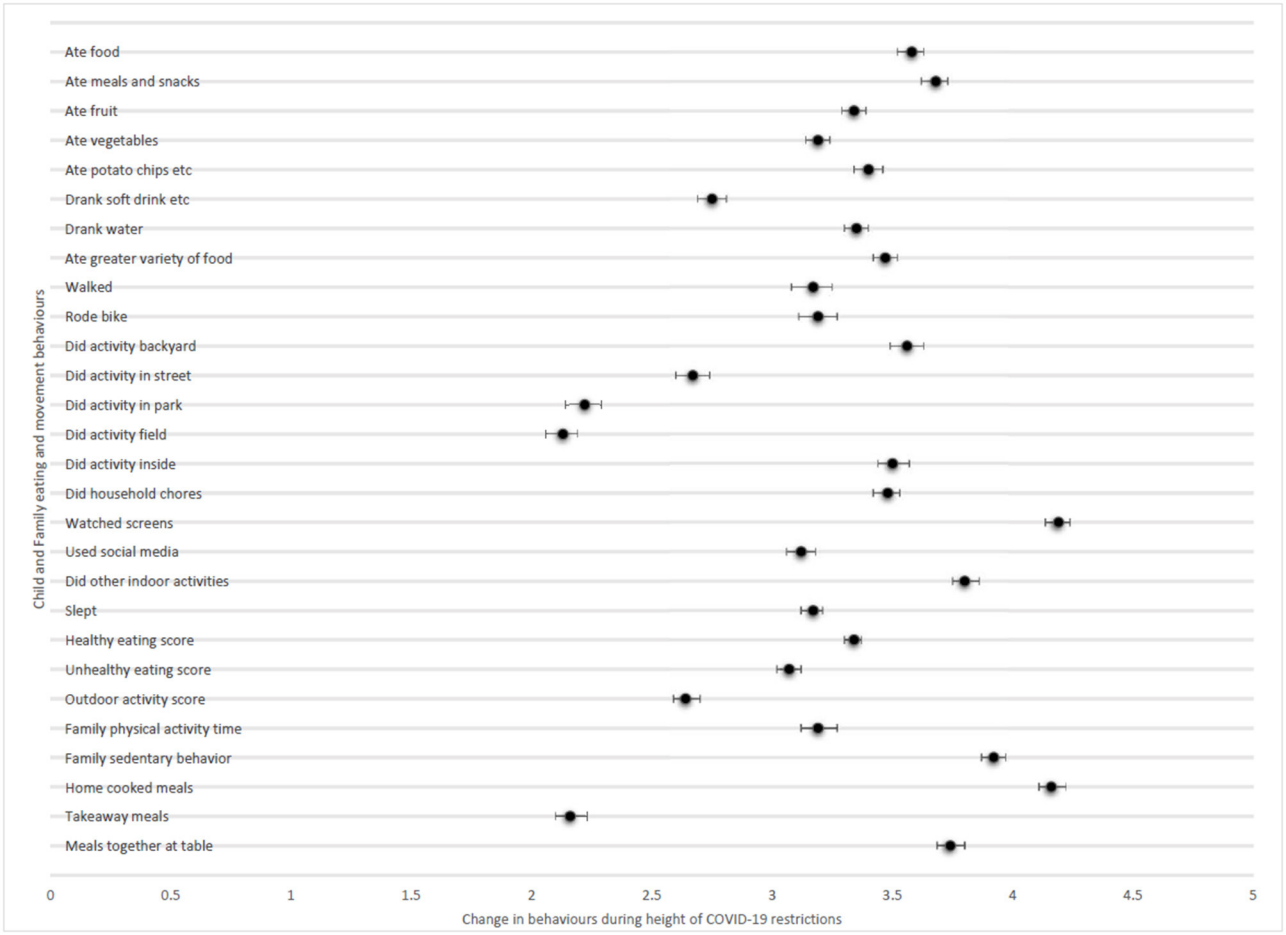
Parent-reported changes in child and family eating and movement behaviors (attached file). Child and family movement behaviors and family eating behaviors: A lot less = 1, a lot more = 5. Child eating behaviors: Totally disagree = 1, totally agree = 5.

Figure 1 displays the parent-reported changes in child and family eating and movement behaviors. Some physical activities remained relatively stable throughout the height of COVID-19 restrictions compared to before, but others, such as physical activity, sport or play in the backyard/garden (3.56/5.00), inside physical activity (3.51/5.00), household chores (3.48/5.00), and other indoor activities (3.80/5.00) increased slightly. There appeared to be a very small increase in sleep (3.17/5.00), but a notable increase in sedentary screen use (4.18/5.00). In regard to eating behaviors, there were small increases in amount of food consumed (3.58/5.00) and the amount of meals and snacks eaten (3.69/5.00). On a positive note, there was a slight increase in the variety of foods eaten (3.47/5.00). When considering scores which combined several questions, there appeared to be very little change to outdoor physical activity overall (2.65/5.00), but a small increase in walking and bike riding (3.18/5.00). Scores also indicated that children were eating more (3.63/5.00), which included an increase in healthy (3.34/5.00) and unhealthy (3.07/5.00) foods. Similar to child behavior, family time spent in sedentary screen time increased (3.91/5.00), and family physical activity increased slightly (3.20/5.00). There was an increase in home cooked meals (4.16/5.00) and eating meals together at the dinner table (3.75/5.00), and a decrease in takeaway meals consumed (2.16/5.00).

### Differences in Changes by Age Group

Changes by age group are displayed in Table 3. There was a significant difference between younger and older age groups in regard to several child and family behaviors. There were significantly greater reported child behavior increases in walking and bike riding (t = −2.041, *p* = 0.041), household chores (t = −4.331, *p* < 0.001), screen time (t = −6.015, *p* < 0.001), social media (t = −5.506, *p* < 0.001), other indoor activities (t = −2.491, *p* < 0.001) and sleep (t = −3.719, *p* < 0.001) in the 5–12 years age group compared to the <5 years age group. Whereas, there was significantly more indoor activity in the <5 years age group compared to the older age group (t = 2.582, *p* = 0.010). Children in the 5–12 years age group also had higher levels of increased food consumption (t = −8056, *p* = 0.000), including both healthy (t = 4.034, *p* = 0.000) and unhealthy (−3.669, *p* = 0.000) foods compared to the younger age group. There were also significantly greater changes in the 5–12 years group compared to the <5 years group in regard to the following reported family behaviors: sedentary time (t = −4.289, *p* = 0.000), home cooked meals (t = −2.660, p = 0.008) and meals eaten at the dinner table (t = −3.719, *p* = 0.000) while increases in takeaway food consumption were significantly more in the <5 years age group compared to the 5–12 year old age group (t = 3.071, *p* = 0.002).

**Table 3:** Changes in child and family eating and movement behaviors stratified by age group.

**Variable**	** <5 years (*n* = 374)**	**5–12 years (*n* = 943)**	**All (*n* = 1,317)**	**Mean difference (95% CI)**	**t**	***p*-Value**
**Change in child eating and movement behaviors**						
Walking and bike riding	3.04 (1.415)	3.23 (1.460)	3.17 (1.450)	−0.179 (−0.350– −0.007)	−2.041	**0.041**
Household chores	3.31 (0.892)	3.55 (0.974)	3.48 (0.947)	−0.239 (−0.348– −0.131)	−4.331	**<0.001**
Sedentary screen time (TV etc)	3.94 (0.964)	4.27 (0.897)	4.18 (0.929)	−0.333 (−0.441– −0.224)	−6.015	**<0.001**
Social media	2.89 (0.909)	3.22 (1.166)	3.12 (1.108)	−0.326 (−0.446– −0.212)	−5.506	**<0.001**
Other indoor activities	3.69 (1.032)	3.85 (1.030)	3.80 (1.033)	−0.155 (−0.277– −0.33)	−2.491	**0.013**
Sleep	3.04 (0.759)	3.22 (0.909)	3.17 (0.872)	−0.181 (−0.276– −0.085)	−3.719	**<0.001**
Outdoor activities score	2.63 (1.015)	2.66 (1.074)	2.65 (1.057)	−0.030 (−0.152– 0.927)	−0.464	0.643
Eat more score	3.33 (0.806)	3.75 (0.944)	3.63 (0.926)	−0.417 (−0.518– −0.315)	−8.056	**<0.001**
Healthy eating score	3.22 (0.619)	3.38 (0.684)	3.34 (0.670)	−0.158 (−0.234– −0.081)	−4.034	**<0.001**
Unhealthy eating score	2.93 (0.868)	3.13 (0.911)	3.07 (0.903)	−0.197 (−0.303– −0.092)	−3.669	**<0.001**
**Change in family eating and movement behaviors**						
Time in physical activity	3.10 (1.350)	3.24 (1.365)	3.20 (1.362)	−0.141 (−0.301– 0.020)	−1.714	0.087
Time in sedentary behavior	3.73 (1.019)	3.99 (0.942)	3.91 (0.971)	−0.258 (−0.377– −0.140)	−4.289	**<0.001**
Home cooked meals	4.05 (1.016)	4.20 (0.946)	4.16 (0.969)	−0.155 (−0.269– −0.041)	−2.660	**0.008**
Takeaway foods	2.32 (1.208)	2.10 (1.197)	2.16 (1.204)	0.223 (0.080– 0.365)	3.071	**0.002**
Meals at dinner table	3.60 (0.948)	3.81 (0.987)	3.75 (0.980)	−0.215 (−0.329– −0.102)	−3.719	**<0.001**

*Bold denotes that the p values are < 0.05*.

### Differences in Changes by Socioeconomic Status

In regard to differences in eating and movement behaviors according to socioeconomic status, there was a significant difference between families in lower and higher socioeconomic areas in regard to reported child unhealthy eating, with children in higher socioeconomic areas having lower unhealthy eating scores (t = 2.739, p = 0.006). There were also significant differences between socioeconomic status and walking/bike riding (t = −7.521, *p* < 0.001), and outdoor activity score (t = –5.415, *p* < 0.001), with children from higher socioeconomic areas having higher levels of reported improvement. There was also a significant difference between groups in relation to family sedentary time, with households in lower socioeconomic areas spending more time sedentary (t = 2.313, *p* = 0.021). Families in lower socioeconomic areas also had more meals together at the table compared to families from higher socioeconomic areas (t = 2.538, *p* = 0.011), while children from higher socioeconomic areas did more household chores than children from lower socioeconomic areas (t = −4.727, *p* < 0.001).

### Differences in Movement Behaviors by Type of Dwelling

In regard to differences in movement behaviors according to type of dwelling, there was a significant difference between living in a house compared to both an apartment/unit and townhouse/villa/duplex/other in regard to walking and bike riding [F (2, 1306) = 37.965, *p* < 0.001], and outdoor activity score [F (2, 1304) = 27.380, *p* = 0.000], with children residing in a house participating in more of these activities than those residing in other dwellings. There was a significant difference between living in a house and an apartment/ unit or townhouse/villa/duplex/other in regard to household chores [F (2, 1308) = 5.426, *p* = 0.005], with children in houses performing more. There was a significant difference between families living in houses and apartments/units in regard to family time spent in physical activity [F (2, 1309) = 7.444, *p* = 0.001]. There was also a significant difference between dwelling type in regard to change in child social media use [F (2, 1305) = 4.378, *p* = 0.013], with children residing in a house using social media more than those living in an apartment/unit. There was no significant difference between dwelling type in regard sleep, screen time use, or other quiet indoor activities.

### Changes Parents Would Like to Maintain

Of the 1,371 parents who completed the survey 1,363 parents responded to the open-ended question on the behaviors that they would like to maintain “more or less” of after lockdown. The majority of behaviors that parents reported were in regard to behaviors that their child or family were doing more of during lockdown that they would like to see continue (*n* = 855, 63%), with a smaller proportion of (*n* = 69 parents, 5%) reporting that they wanted to continue doing less of the behaviors that they had reduced during lockdown, 26 parents (2%) reporting there was no change in their behaviors during lockdown, 16 parents (1%) reporting both “more and less” behaviors at the same time and 393 responses (29%) were unrelated to the question. There was an overwhelming response of parents wanting to continue having “family time” (*n* = 420), frequently using those exact words. The second most popular behavior people wanted to continue (*n* = 390) was physical activity, the most popular being: walking and bike riding, followed by bushwalking. Parents commonly reported these two themes together such as “going for family walks” or “bike riding with the family” or “exploring the outdoors/bushwalking with the family.” The other two behaviors that were mentioned frequently were “home cooking” and “eating family meals together” (*n* = 153) and doing hobbies (*n* = 135), including puzzles, board games, reading, gardening, and craft. Parents also reported that they enjoyed having time to relax and not rushing around (*n* = 55). They reported that they liked having healthy hygiene habits (washing hands etc) (*n* = 43) and they reported enjoying having more communication in their family or the family operating more harmoniously with less sibling arguments, more time to talk and more time to play together and get along (*n* = 39). The main behaviors parents reported wanting less of after lockdown were less screen time (*n* = 27) and less family stress/mental health issues (*n* = 22). Parents were also looking forward to less issues related to boredom and misbehavior (*n* = 16).

### Food Security

With reference to the last 12 months, 102 (7.4%) participants reported running out of food. In relation to the period of the height of COVID-19 restrictions in 2020, 914 (66.7%) of respondents reported that their food security stayed the same, whereas 254 respondents (18.5%) stated that they were less able to afford food and 52 respondents (3.8%) stated that they were more able to afford food. Food affordability differed significantly by socioeconomic status, with those from lower socioeconomic areas being more likely to have run out of food in the last 12 months (chi square 26.827, df1, *p* < 0.001), and also being more likely to experience increased food security issues during the height of COVID-19 restrictions (chi square 29.318, df 2, *p* < 0.001).

## Discussion

The aim of this study was to determine changes in eating and movement behaviors in children and families from western Sydney in households with children 0-12-years-of-age during the height of the COVID-19 restrictions in 2020. To our knowledge, this study is the first to examine the early effects of the COVID-19 pandemic on both eating and movement behaviors among Australian children and families. Data were collected during the month of June 2020, subsequent to the first Sydney lockdown to prevent the spread of the SARS COV2 virus. The study will assist in determining the extent of health behavior changes on families resulting from COVID-19 restrictions and guide decision making in regard to initiatives required to address detrimental changes to reduce the risk of long-term health consequences.

Overall, despite the short-term (62 day) period of the first lockdown in Sydney, Australia, the impact of these restrictions appears similar to many of the global changes seen in child and family health behaviors due to lockdowns. The largest impact in this study was noted in relation to family sedentary behaviors, child screen time and social media use. Changes were also seen in relation to eating behaviors, with an increase in meals, snacks and unhealthy foods eaten. There were also some positive food-related changes in that there were more home cooked meals and families were eating more meals together. There was also an increase in consumption of healthy foods. There were changes seen in activity, with small increases overall, but less outdoor activity (with the exception of backyard activity) and more indoor activity.

The increases seen in sedentary behavior, and screen time are not surprising with many parents working from home and children learning remotely. A Canadian study of 1,472 families by Moore et al. (3), which we based our movement questions on, found similar increases in family sedentary time in families with children 5–11-years-of-age (3.78/5.00 compared to 3.91/5.00 in the current study) and child sedentary screen use (4.10/5.00 compared to 4.18/5.00 in the current study). A study by Carroll et al. (4) who investigated changes in child behaviors in 254 Canadian families with children aged 18 months to 5-years-of-age also found changes in screen time, which were related to an increase in online learning. Dunton et al. (5) study for 211 parents of 5–13-year-old children in the US also found increases in sedentary behavior, however most sedentary time was found spent watching television videos or movies. With the increase in screen use for work and learning purposes, this possibly spilled over into more recreational screen time and social media use. It should be noted that not necessarily all this screen time use would be negative, some was essential for educational purposes, and some may have helped in maintaining contact with loved ones. Although there are negative consequences of screen time and social media use (13), for children at this time, it may have assisted in staying connected with friends and family and made a positive contribution to mental wellbeing. However, potential negative consequences should be carefully considered, particularly in the context of long lockdown periods, where prolonged increased screen usage could have a detrimental impact on general health as well as mental wellbeing (14).

Like previous similar studies (4, 6), this study noted a reported increase in eating meals and snacks during the COVID-19 restriction period. Increased food intake during this time could be due to several factors. Spending considerably more time at home indoors and out of regular routines may result in boredom which can lead to increased eating (15). Stress can also be a factor in increased food consumption (16). This may be a result of direct stress of the lockdown on children, not being able to see friends or family, attend school, and difficulty managing remote learning. Anxiety and stress for parents caused by financial pressure, job instability, increased work hours and juggling competing demands could also result in parents overeating and have an indirect on children's food consumption through role modeling. It has been reported that the mental health of 48% of adults and 36% of children in Australia has been affected by COVID-19 (7), so the potential for related overeating is considerable. Increased time on screens and social media may also be a factor in increased food consumption. There is an established relationship between screen time and obesity in children, which is thought to be primarily mediated by food consumption while watching screens and exposure to unhealthy food advertising (17). An Australian study reviewed social media posts of 42 leading food and beverage brands (including fast food, discretionary foods and food delivery services) over 4 months, coinciding with the lockdown period of the current study. It found that almost 80% of these brands capitalized on COVID-19 in their marketing messages, and often these messages related to corporate social responsibility, a strategy which has been demonstrated to increase sales (18). Increases in screen use during this time could therefore have a detrimental impact not only on food intake in the immediate term but also an effect on child weight trajectories.

There appeared to be positive changes in regard to more home cooked meals which was also found by Carroll et al. (4), and in a New Zealand study of adults by Gerritsen et al. (19), half of whom had children under 18 years. Carroll et al. (4) reported that parents expressed interest in involving children in food preparation, and that they would like resources to assist them with this, a strategy which may be useful in future interventions, particularly considering involvement of children in meal planning and preparation has been shown to be predictive of healthier eating (20). While home cooking is generally healthier than takeaway alternatives (21), it should be noted that we did not explore the type of foods being prepared at home. In Gerritsen et al. (19) study, increases in home cooking during lockdown did not necessarily translate to healthier food intake, particularly for households with children. Like Carroll et al. (4), the current study found an increase in the family meals eaten together, possibly due to many parents and children being home together at lunchtime, and many parents being home at dinnertime who may otherwise have long commuting times.

While there was less of an impact on physical activity overall, outdoor activity (other than backyard activity) remained similar, while indoor activity increased. There was a slight increase in walking and bike riding, but less activity in the street and at parks and fields. These findings were similar to Moore et al. (3), though there seemed to be a little more participation in overall child activity in the current study (3.25/5.00) compared to Moore et al. (3) (3.00/5.00), child walking and bike riding (3.18/5.00) compared to Moore et al. (3.00/5.00), and family physical activity (3.20/5.00) compared to Moore et al. (3) (2.72/5.00). These slight differences may be due to differences in the built environments, weather conditions and other differing factors between Australia and Canada. Other studies have reported reductions in child physical activity during lockdowns (4, 5). During the lockdown period in Sydney, there was a reduced variety of opportunities to be active with playgrounds and skateparks closed, and reduction in incidental activities, such as active commuting to school. With the closure of schools (and therefore access to physical education classes and school sport), community sport and other activities, the activity that children undertook during this period was less structured in nature, and it is likely that it was less intensive than activities they participated in prior to lockdown (22). So, although in the current study activity may not have declined overall, it is likely that the activities being undertaken were of lower intensity, which may impact on children's ability to meet the energetic play and moderate to vigorous intensity aspects of the 24-h movement guidelines (23, 24). Parents in the current study reported that their children slept a little more than before, consistent with some studies (3, 8), while other studies have reported sleep was unchanged (4). With children learning from home, it is probable that this increased sleep resulted from children not having to get up as early to leave for school or other activities.

The changes in health behaviors reported in this study have the potential to have a detrimental effect on child weight status. A study in the USA has predicted that based on the impact of school closures for 6 months, child BMI could increase by 0.198 and childhood obesity could increase by 2.373 percentage points. This is solely based on reduction in activity from children not being able to attend school physical education classes and does not factor in any dietary intake changes (25). At the time of writing in 2021, NSW has experienced a recent outbreak resulting in a further lockdown period. Schools closed from June until October 2021. In addition to the initial 4-week closure in 2020, this would amount to a total period of 5 months. While predictions made by An et al. (25) are based on USA data, it is possible that these changes would be similar for children in NSW, and potentially much worse in the state of Victoria, where schools have been closed intermittently for a total of over 8 months during 2020 and 2021.

This study found that children from lower socioeconomic areas reported less physical activity and ate less healthily than those from higher socioeconomic areas during the COVID-19 restrictions. Further, children from lower socioeconomic areas reported an increase in sedentary behavior compared to those from higher socioeconomic areas. It is well-recognized that health inequity is impacted by social determinants such as access to health care, education, employment opportunities, adequate income and affordable housing (26). Overweight and obesity rates in NSW are higher in children from lower socioeconomic backgrounds than children from higher socioeconomic backgrounds. Cardiorespiratory fitness rates have also been reported to be lower in children from lower socioeconomic backgrounds (27). Furthermore, in lower socioeconomic areas, there are generally less safe places to exercise, and less access to greenspaces, making it more challenging for people to be active (22). Cost associated with sporting activities may also contribute to this lower level of fitness. Targeted policies and programmatic strategies are therefore required to ensure the gap between lower and higher socioeconomic families is not exacerbated and widened as a consequence of the pandemic.

Children living in a house increased their overall outdoor activity significantly more than children living in other types of housing. This was also the case in regard to walking and bike riding specifically. Likewise, Moore et al. (3) also found that children that lived in detached houses did more outdoor physical activity and spent more time walking and bike riding. Similarly, Mitra et al. (6) found that residing in a house was correlated with increased outdoor activities. Previous research has demonstrated that children are more active when they are outside than inside (28). Access to more outdoor space for those that live in houses compared to other housing types may account for some of the differences seen in the current study. This finding reinforces the need for judicious urban planning in areas with high-density housing to ensure that there is nearby access to facilities such as greenspaces and walking and cycling tracks to enable families that do not have access to ample outdoor spaces on their property to be active (29). This disparity in outdoor activity may also have been exacerbated by the closure of playgrounds and skateparks at the height of restrictions.

While most parents reported that their children's sedentary behavior increased, older children (5–12-years-of-age) were significantly more affected. Although sedentary behavior increases as children get older (30), the COVID-19 pandemic may be accelerating these changes. Greater increases in sedentary behavior during lockdown in children aged 9–13-years compared to younger children were also noted by Dunton et al. (5) in the USA. Patterns observed in parents' perceptions of changes in children's sedentary behavior further underscore the heightened risk of the pandemic on older children. It is concerning that older children may adopt new behavioral habits of physical inactivity during the pandemic that are extremely difficult to change. The development of healthy habits in childhood needs to be a high priority with particular attention paid to screen time. Previous and current health promotion programs to date focus on educating parents on screen time guidelines, but perhaps like similar public health behaviors (physical activity and healthy eating) broader environmental strategies need to be considered to help regulate screens outside the home, such as in early childhood settings and primary schools and a higher priority in general is given to movement behaviors in schools and childcare settings.

A report by an Australian food charity in 2021 reported that 28% of Australians were food insecure over the past 12 months, and 38% of those who are food insecure had not experienced food insecurity prior to the COVID-19 pandemic (31). While this proportion is much higher than the 7% that reported that they had run out of food in the last 12 months in the current study, 18.5% reported that they were less able to afford food during the height of the restrictions. It should also be noted that the sample was a higher socioeconomic demographic compared to the general western Sydney population, with 75% of the study sample living in the most advantaged postcodes (6–10 top deciles). It is therefore possible that the actual percentage could be larger across the western Sydney population. With the continued lockdown periods that have occurred since our study was conducted, it is also likely that the situation has further deteriorated since.

Prior to COVID-19, in New South Wales, many children were not meeting guidelines for healthy eating and physical activity (32). The negative impact of COVID-19 restrictions is of public health concern as there is the potential for exacerbation of existing unhealthy behaviors in children and families and danger that these habits could become permanently entrenched, increasing the risk of obesity, diabetes and cardiovascular disease in later life. While there has been a plateauing of childhood overweight and obesity rates in NSW in recent years (albeit at high rates of around 23% of 5–16-year-olds in 2019) (33), behavior changes seen during the COVID-19 lockdown could threaten this progress and childhood obesity rates could again begin to increase. Currently, there are no Australian data available on child post-COVID weight status. It is important that overweight and obesity rates are monitored closely during COVID recovery to determine if rates begin to increase. During lockdown, public health messaging has concentrated on infection control and social distancing, with little focus on healthy eating and physical activity and sedentary behavior. It is critical that COVID-19 communications are balanced with health promotion messaging. Programmatic and policy strategies geared toward reducing sedentary behavior, increasing moderate-vigorous intensity physical activity and improving eating behaviors are essential as Australia navigates its way through and beyond the pandemic. Upstream approaches are also essential to ensure that environmental factors influencing such behaviors are addressed. The World Health Organization recommended in their Report of the Commission on Ending Childhood Obesity in 2016 that several initiatives be implemented, including regulating unhealthy food marketing, improving food labeling, taxation of sugar sweetened beverages and creating supportive environments in settings where children gather (34). These recommendations have become even more pertinent in the post-COVID recovery period.

### Study Limitations and Strengths

One of the strengths of this study was the large sample size of families from western Sydney. Our study had some limitations. Data were self-reported, so there may be a degree of social desirability bias in the responses. The questions also relied on participants recalling what happened prior to and during the lockdown. Participants were not necessarily representative of the western Sydney population as a whole. It may have been that those who completed the survey were more interested in their child's lifestyle behaviors than those that chose not to participate.

## Conclusion

This study provides insights into child and family lifestyle changes during the COVID-19 pandemic in western Sydney, NSW, Australia. Even with the lower level and shorter time-period of restrictions in NSW, Australia in 2020 compared to the rest of the world, there appeared to be notable parent-reported impacts on child and sedentary behaviors and eating behaviors, and child outdoor physical activity. Some of these impacts were more substantial in older children and families residing in areas of higher socioeconomic disadvantage. These disparities highlight the need to enact policies and programs to support families in developing healthy lifestyle habits during COVID recovery.

## Data Availability Statement

The datasets presented in this article are not readily available because the datasets presented in this article are not readily available because participants have not provided consent for future use of data. Requests to access the datasets should be directed to JM, Janelle.McNicholas@health.nsw.gov.au.

## Ethics Statement

This study involving human participants was reviewed and approved by Western Sydney Local Health District Human Research Ethics Committee. Written informed consent for participation was not required for this study in accordance with the national legislation and the institutional requirements.

## Author Contributions

JM: research concept and design, preparing research proposal, conducting qualitative data analysis, and writing the manuscript. MH: research concept and design, preparing research proposal, conducting quantitative and qualitative data analysis, and writing the manuscript. SH and SM: preparing research proposal, dissemination of survey, data cleaning, some data analysis, and reviewing and editing of manuscript. JP: preparing research proposal, dissemination of survey, and reviewing and editing of manuscript. All authors contributed to the article and approved the submitted version.

## Conflict of Interest

The authors declare that the research was conducted in the absence of any commercial or financial relationships that could be construed as a potential conflict of interest.

## Publisher's Note

All claims expressed in this article are solely those of the authors and do not necessarily represent those of their affiliated organizations, or those of the publisher, the editors and the reviewers. Any product that may be evaluated in this article, or claim that may be made by its manufacturer, is not guaranteed or endorsed by the publisher.
